# Dysgonomonas capnocytophagoides Bacteremia in a Patient With Stage IV Colon Adenocarcinoma

**DOI:** 10.7759/cureus.16381

**Published:** 2021-07-14

**Authors:** Sarah E Schall, Dana M Blyth, Shannon L McCarthy

**Affiliations:** 1 Internal Medicine, Brooke Army Medical Center, Fort Sam Houston, USA; 2 Infectious Diseases, Walter Reed National Military Medical Center, Bethesda, USA; 3 Infectious Diseases, Mike O'Callaghan Military Medical Center, Las Vegas, USA

**Keywords:** dysgonomonas capnocytophagoides, instrinsic multidrug resistance, molecular identification

## Abstract

*Dysgonomonas capnocytophagoides* bacteremia is a rare clinical entity described in only five case reports. Difficulties in the identification and intrinsic multidrug resistance (MDR) of the organism make diagnosis and treatment challenging. We present a case of *D. capnocytophagoides* bacteremia which highlights the diagnostic and treatment challenges posed by this organism. The case also contributes to the nascent understanding of the clinical profile of patients with *D. capnocytophagoides* infection and the antimicrobial susceptibility of the organism.

A 56-year-old male with advanced colon adenocarcinoma on palliative fluorouracil, leucovorin, and irinotecan (FOLFIRI) presented with abdominal pain. He had been discharged recently following an ICU admission for neutropenic fever with diarrhea and polymicrobial bacteremia resulting in sepsis. Diarrhea resolved during hospitalization. Mediport was retained, surveillance blood cultures remained negative, and he completed 14 days of levofloxacin. Upon readmission for abdominal pain, vital signs were normal and neutropenia had resolved. A Gram-negative rod grew from blood cultures drawn peripherally and from the port with no differential time-to-positivity. Multiple testing platforms were used in an attempt to identify the organism, to include the VERIGENE® Gram-negative blood culture test, matrix-assisted laser desorption ionization-time of flight (MALDI-TOF) mass spectrometry, VITEK ® 2 GN ID, and the Thermo Scientific™ RapID™ NH System (Thermo Scientific, Waltham, MA). Test results from all platforms were either inconclusive or contradictory in their identification of the organism, making the determination of appropriate treatment difficult. Given inconsistent results on multiple testing platforms, the isolate was sent for whole genome sequencing (WGS). Additional workup performed during the hospitalization included a diagnostic paracentesis without evidence of spontaneous bacterial peritonitis, transesophageal echocardiogram without evidence of infective endocarditis, and dental evaluation without evidence of the infectious source. Abdominal CT showed nonspecific terminal ileitis. He was treated for presumed HACEK bacteremia and was transitioned from piperacillin-tazobactam to ceftriaxone to complete a two-week course at hospital discharge. He also received a seven-day course of doxycycline for concomitant, mild lower extremity cellulitis which resolved during hospitalization. Ultimately, antimicrobial susceptibility testing which resulted following discharge was not consistent with the HACEK organism. Testing demonstrated resistance to multiple antimicrobials including ceftriaxone, as well as susceptibility to trimethoprim-sulfamethoxazole (TMP/SMX). WGS ultimately identified the organism as *D. capnocytophagoides*. Despite ceftriaxone resistance, he reported feeling well at follow-up with negative surveillance blood cultures.

This patient shares several features with the few patients previously identified with *D. capnocytophagoides* bacteremia, including malignancy, recent neutropenia, and presumed gastrointestinal source. As in the small number of prior reported cases, the organism was difficult to identify leading to delay in diagnosis and treatment. The case demonstrates the importance of critical thinking in the face of contradictory test results. Additionally, based on susceptibility profiles described in prior literature, we suspect doxycycline treated his bacteremia.

## Introduction

*Dysgonomonas capnocytophagoides* is a Gram-negative coccibacilli and facultative anerobe that has only been recently recognized as a human pathogen [1]. Bacteremia secondary to the organism [previously categorized as dysgonic fermenter-3 (DF-3) by the Centers for Disease Control and Prevention (CDC) prior to its recognition as a distinct species) is a rare clinical entity described in only five case reports, all occurring in immunocompromised patients or those with malignancies [2-4]. The organism has also been isolated from the stool of immunosuppressed patients [5] and available data suggest that it is part of the normal gastrointestinal flora, although it has also been isolated in urine [6] and from skin abscesses in patients with diabetes [7]. Prior case reports describe difficulty in identification of the organism and intrinsic multidrug resistance (MDR) which make antimicrobial selection challenging. We present a case of successfully treated *D. capnocytophagoides* bacteremia which highlights the challenges in identification and appropriate antimicrobial selection posed by this organism and contributes to the current understanding of *Dysgonomonas* infection which is limited by the organism's rarity.

## Case presentation

The patient is a 56-year-old male with stage IV colon adenocarcinoma status post hemicolectomy with known liver metastases and recurrent malignant ascites on palliative chemotherapy with fluorouracil, leucovorin, and irinotecan (FOLFIRI). He presented to the emergency department (ED) with worsening ascites and abdominal pain. He had been discharged recently following ICU admission for neutropenic sepsis, diarrhea, and polymicrobial bacteremia with *Klebsiella pneumoniae*, *Escherichia coli*, and *Streptococcus mitis* presumed secondary to a gastrointestinal source. Stool cultures and *Clostridium difficile* testing were negative during his prior admission and his diarrhea resolved. His Mediport was retained, surveillance blood cultures were negative, and he completed 14 days of levofloxacin.

He was readmitted for abdominal pain and increasing abdominal girth. His vital signs were normal and neutropenia had resolved. Blood cultures were obtained on the day of admission for work-up of possible spontaneous bacterial peritonitis in an immunocompromised host. A Gram-negative rod was isolated from anerobic blood cultures drawn both peripherally and from the port without differential time-to-positivity. No identification was reported by VERIGENE® (Luminex Corporation, Austin, TX) Gram-negative blood culture test or matrix assisted laser desorption ionization-time of flight (MALDI-TOF) mass spectrometry despite multiple attempts. The VITEK ® 2 GN ID (bioMerieux, Marcy-l'Etoile, France) reported Sphingomonas paucimobilis. Thermo Scientific™ RapID™ NH System determined the isolate to be Hemophilus parainfluenzae then on repeat attempt Aggregatobacter aphrophilus, but all had contradicting biochemical tests concerning for inaccurate identification. The isolate was sent to the Multidrug resistant organism Repository and Surveillance Network (MRSN) for whole genome sequencing (WGS) on an Illumina Miseq Benchtop sequencer (Illumina, San Diego, CA).

During his hospital course, the patient remained afebrile. Paracentesis was not consistent with spontaneous bacterial peritonitis. There was a concern for a HACEK organism, a collection of fastidious Gram-negative bacilli with long incubation periods when grown on traditional, non-automated blood culture systems that are associated with endocarditis. The transesophageal echocardiogram showed no evidence of endocarditis and dental evaluation was negative for an infectious source. Abdominal and pelvic CT showed nonspecific terminal ileitis felt to be related to recent diarrheal illness. The patient’s diarrhea had not recurred. Incidentally, he received a seven-day course of doxycycline, started on admission, for mild lower extremity cellulitis which resolved during hospitalization. On discharge, he was transitioned from piperacillin-tazobactam, started on admission with seven days of total treatment, to a two-week course of ceftriaxone for presumed HACEK bacteremia while awaiting susceptibilities and final identification.

Ultimately, antimicrobial susceptibility testing revealed conflicting results for a HACEK organism: resistance to amikacin, ceftriaxone, levofloxacin, meropenem, ampicillin-sulbactam, and cefepime and susceptibility to trimethoprim-sulfamethoxazole (TMP/SMX). Despite ceftriaxone resistance, he reported feeling well at follow-up with negative surveillance blood cultures from both port and peripheral sources. The patient continued to show no signs of infection on follow-up.

Analysis of the WGS data with Kraken later identified the organism as *D. capnocytophagoides*. WGS ultimately showed no known antibiotic resistance genes, but there is currently insufficient data on *D. capnocytophagoides* to identify mutations that confer resistance. The sequence for this strain (*D. capnocytophagoides* MRSN 571793) has been deposited in the NCBI database under Genbank accession number NZ_SOML00000000.

## Discussion

This patient shares several features with the few patients previously identified with *D. capnocytophagoides* bacteremia. All existing case reports of *D. capnocytophagoides* bacteremia have occurred in patients with malignancy, and the majority were immunocompromised. Three patients had acute leukemia, were actively receiving chemotherapy, and were neutropenic at the time of diagnosis [4, 8]. Of these, one presented with a diarrheal illness and two with fever. Another patient had diabetes mellitus and pancreatic cancer and presented with symptoms of cholangitis, although the authors did not consider *D. capnocytophagoides* to be the likely causative organism of the cholangitis due to the patient’s profound clinical improvement following treatment with cefoperazone-sulbactam to which the Dysgonomonas was not susceptible [2]. The final patient had hepatocellular carcinoma and diabetes mellitus and had recently undergone radiofrequency ablation of the liver [3]. This patient grew the organism from both blood and liver abscess cultures. Our patient is the first case of *D. capnocytophagoides* bacteremia described in a patient with adenocarcinoma of the colon. Additionally, while our patient was not neutropenic at the time of his diagnosis, he had a recent prolonged period of neutropenia. Interestingly, although *D. capnocytophagoides* has been most frequently isolated from the stool, only three of the six patients described, including ours, had a diarrheal illness around the time of their diagnosis with bacteremia. These cases are consistent with the presumed gastrointestinal origin of the bacterium, although its exact mechanism of pathogenicity has not been fully elucidated.

In addition to the presence of malignancy, recent antimicrobial treatment is a common factor in several of the patients described. Four of them, including our patient, had recently received or were actively receiving broad-spectrum antimicrobial coverage, and one additional patient had received a dose of prophylactic cefazolin two days prior to presentation. The four patients on broad-spectrum coverage had recent or concurrent bacteremia with other organisms at the time of diagnosis: one with Prevotella, one with *Streptococcus agalactiae*, and one who like our patient had polymicrobial bacteremia with *E. coli* and *K. pneumoniae*. The nature of these co-infected species again supports that *D. capnocytophagoides* is part of the gastrointestinal flora. Additionally, recent antimicrobial exposure may represent a risk factor for *D. capnocytophagoides* infection, although the small sample size does not allow for a definitive determination. Interestingly, a case series that examined stool isolates of *D. capnocytophagoides* from eight immunosuppressed patients noted that four of the eight had recent antimicrobial exposure prior to collection of the stool sample [5]. This same series presented a case of asymptomatic carriage of the organism in a patient without recent antimicrobial exposure. These findings raise the question of whether the organism’s pathogenesis may be linked to suppression of the colonization resistance of typical gastrointestinal flora via recent antimicrobial usage. Further study would be required to make this determination. This would likely require concerted effort given the difficulty of cultivating and identifying the organism in vitro.

A review of prior cases also demonstrates similar antimicrobial susceptibility profiles. Existing data demonstrate resistance to ampicillin, cephalosporins, carbapenems, aminoglycosides and fluoroquinolones and susceptibility to tetracyclines, TMP-SMX, and chloramphenicol, which is consistent with the susceptibility data in our case. Multiple prior case reports describe the failure of broad-spectrum treatment with piperacillin-tazobactam and imipenem which is consistent with the organism's known intrinsic MDR. This raises the question of whether broad-spectrum antibiotic coverage in these immunocompromised patients is selecting for the growth of *D. capnocytophagoides* due to its MDR. Successful treatment of bacteremia has been described with TMP-SMX and cefoperazone-sulbactam. One case report describes the resolution of bacteremia with the patient’s immune reconstitution despite treatment with only antibiotics with in vitro resistance. Given the susceptibility profile of the organism in our case, we suspect that it was treated by the doxycycline that the patient received for cellulitis. Although there is no prior report of *D. capnocytophagoides* bacteremia being treated with doxycycline, one series examining stool isolates reports an AIDS patient with diarrhea and positive stool culture for *D. capnocytophagoides* treated with doxycycline 100 mg twice daily by mouth with subsequent negative stool culture and improvement in diarrhea [5].

A final common feature in these cases is the delay in diagnosis resulting from difficulty in identifying the organism. In a milieu of increasingly complex and more widely available methods of pathogen identification, it is important for every clinician to have a solid understanding of the capabilities of the most common systems, as well as their inherent limitations. In our case, several rapid molecular diagnostic assays including the VERIGENE® and the MALDI-TOF were unable to provide identification. VERIGENE® is limited to the identification of nine common GNR pathogens and several beta-lactam resistance genes via multiplex assay and is therefore not useful in identifying uncommon pathogens [9]. The MALDI-TOF, which employs mass spectrometry to provide characteristic information about the biochemical composition of a specimen [10], uses comparison to a database of known biochemical profiles, which again renders it less useful in the identification of rare organisms. The VITEK ® 2 GN ID also identifies microorganisms based on biochemical profile and reports an accuracy rate of 88.5% at three hours [11], again demonstrating the low discriminatory ability for some uncommon pathogens and resulting in incorrect identification in our case. Finally, the Thermo Scientific™ RapID™ NH System, which relies on detection of specific substrates that are produced by microbial degradation, identified two different pathogens in this case, neither of which was the pathogen ultimately identified by WGS [12]. Of note, *D. capnocytophagoides* is a glucose-fermenting facultative anerobe, and like Aggregatobacter aphrophilus it is oxidase and catalase negative, likely accounting for the misidentification in this case. The conflicting biochemical results raised the healthcare team’s suspicion for an uncommon pathogen and spurred further investigation with WGS with Kraken [13]. While sequencing provides the most accurate identification, it is limited in its clinical efficacy by high turnaround time and expense. Additionally, as in this case, WGS is not able to identify antibiotic resistance due to point mutations in the case of rare pathogens such as *D. capnocytophagoides* due to lack of data on wildtype genome. While complex laboratory methods are increasingly used in clinical decision making, it is vital that practitioners employ a high level of critical thinking when interpreting results, especially when they are contradictory. Furthermore, it is important to continue to expand existing databases to facilitate the diagnosis of rarer pathogens.

## Conclusions

This patient shares several features with the few patients previously identified with *D. capnocytophagoides* bacteremia, including malignancy, recent neutropenia, presumed gastrointestinal source, and delayed identification. It is, however, the first case described in a patient with adenocarcinoma of the colon. Recent antimicrobial exposure also appears to represent a risk factor for *D. capnocytophagoides* infection, although a small sample size makes this difficult to determine definitively. Existing case reports demonstrate similar susceptibility profiles consistent with the organism's intrinsic MDR. Based on susceptibility profiles described in prior literature, we suspect doxycycline treated this patient's bacteremia. Successful treatment of *D. capnocytophagoides* bacteremia with doxycycline has not been previously described. Delayed identification in *D. capnocytophagoides* cases demonstrates the necessity for practitioners to employ a high level of critical thinking when interpreting results, as well as the importance of continued expansion of existing databases to allow diagnosis of rare pathogens.
